# Epigenetic Deregulation of MicroRNAs in Rhabdomyosarcoma and Neuroblastoma and Translational Perspectives

**DOI:** 10.3390/ijms131216554

**Published:** 2012-12-05

**Authors:** Paolo Romania, Alice Bertaina, Giorgia Bracaglia, Franco Locatelli, Doriana Fruci, Rossella Rota

**Affiliations:** 1 Department of Pediatric Hematology-Oncology, Ospedale Pediatrico Bambino Gesù, IRCCS, Piazza S. Onofrio 4, Roma 00165, Italy; E-Mails: paolo.romania@opbg.net (P.R.); alice.bertaina@opbg.net (A.B.); giorgia.bracaglia@opbg.net (G.B.); franco.locatelli@opbg.net (F.L.); 2 Department of Pediatric Sciences, University of Pavia, Corso Strada Nuova 65, Pavia I-27100, Italy

**Keywords:** microRNA, rhabdomyosarcoma, neuroblastoma, epigenetics, differentiation, Polycomb proteins, DNA methylation, histones

## Abstract

Gene expression control mediated by microRNAs and epigenetic remodeling of chromatin are interconnected processes often involved in feedback regulatory loops, which strictly guide proper tissue differentiation during embryonal development. Altered expression of microRNAs is one of the mechanisms leading to pathologic conditions, such as cancer. Several lines of evidence pointed to epigenetic alterations as responsible for aberrant microRNA expression in human cancers. Rhabdomyosarcoma and neuroblastoma are pediatric cancers derived from cells presenting features of skeletal muscle and neuronal precursors, respectively, blocked at different stages of differentiation. Consistently, tumor cells express tissue markers of origin but are unable to terminally differentiate. Several microRNAs playing a key role during tissue differentiation are often epigenetically downregulated in rhabdomyosarcoma and neuroblastoma and behave as tumor suppressors when re-expressed. Recently, inhibition of epigenetic modulators in adult tumors has provided encouraging results causing re-expression of anti-tumor master gene pathways. Thus, a similar approach could be used to correct the aberrant epigenetic regulation of microRNAs in rhabdomyosarcoma and neuroblastoma. The present review highlights the current insights on epigenetically deregulated microRNAs in rhabdomyosarcoma and neuroblastoma and their role in tumorigenesis and developmental pathways. The translational clinical implications and challenges regarding modulation of epigenetic chromatin remodeling/microRNAs interconnections are also discussed.

## 1. Introduction

Epigenetic chromatin remodeling plays a pivotal role in normal mammalian development and post-natal tissue homeostasis. Indeed, lineage specification and cellular differentiation, which underlie embryo development and morphogenesis from a single pluripotent stem cell, are epigenetically regulated processes. The final result is the “plasticity” of an individual genotype that, through the activation of molecular cascades, timely and sequentially controlled, produces different phenotypes in response to different microenvironments. In the last 10 years, special attention has been paid to the non-protein coding portion of the genome such as non-coding small RNAs, among which are microRNAs (miRNAs), considered to be major regulators of developmental pathways [1–8]. Of note, chromatin remodeling and miRNA pathways have been shown to be interconnected and able to regulate each other. To date, it is recognized that the deregulation of the epigenetic- and miRNA-dependent control of gene expression underlies tumorigenesis.

Rhabdomyosarcoma (RMS) and neuroblastoma (NB) are pediatric cancers derived from cells sharing molecular features of skeletal muscle and neuronal progenitors, respectively, blocked at different stages of differentiation. It has been clearly demonstrated that deregulation of developmental pathways plays a major role in both tumors.

The present review will focus on current knowledge about miRNAs deregulated in RMS and NB by epigenetic modifications. Their role in developmental pathways and RMS and NB tumorigenesis will be highlighted. Moreover, the translational implications and challenges of miRNAs modulation in these pediatric tumors will be discussed.

## 2. What the Term “Epigenetics” Means

The term “epigenetics” describes cellular modifications caused by mechanisms other than DNA sequence variations that can be heritable and modified by environmental stimuli [9]. Therefore, in the present review, we will refer to epigenetics to describe the chemical reactions of chromatin that affect its accessibility in order to switch “on” and “off” gene expression.

**Figure 1:**
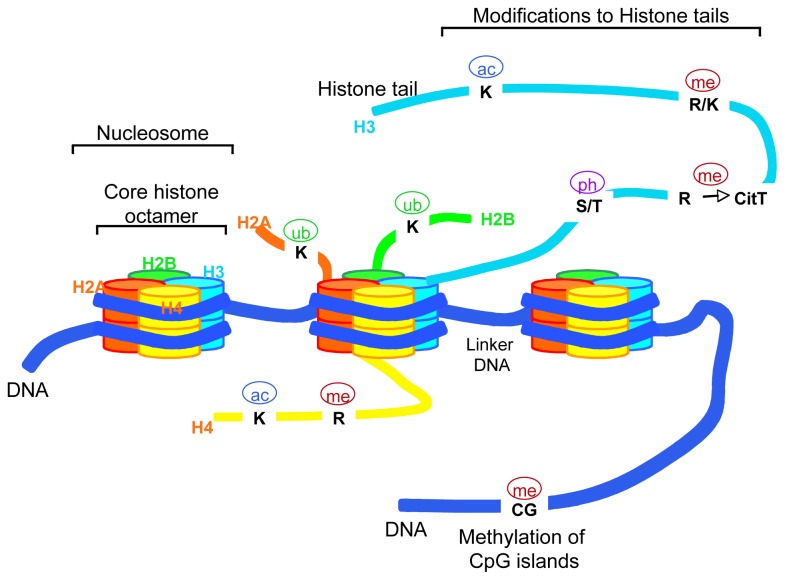
Schematic representation of chromatin structure. Eukaryotic DNA is wrapped around core histone proteins (histone octamer: H2A, H2B, H3 and H4) to form compact chromatin structures termed nucleosomes. Covalent modifications to histones (on histone tails) involve amino acidic residues (Lysine (K), Arginine (R) Serine (S) and Tyrosine (T)) that can be acetylated (ac), methylated (me), phosphorylated (ph) and/or ubiquitinilated (ub). CpG islands on DNA can be methylated. These post-translational modifications result in the epigenetic regulation of gene expression.

To fit within the nucleus of eukaryotic cells, short lengths of DNA are wrapped around an octamer of histone proteins (two copies of core histones H2A, H2B, H3 and H4) forming the basic units of chromatin termed “nucleosomes” (Figure 1).

Higher-order organizations are formed by further compaction of chromatin structure. The degree of packaging influences the chromatin accessibility to transcriptional-regulatory complexes switching a gene “on” or “off.” Therefore, the “epigenome” encompasses genetic information that is the result of multiple processes involving chromatin modifications such as DNA methylation, histone proteins modification, histone variants replacement, nucleosome repositioning and mechanisms involving non-coding RNAs function. All these epigenetic modifications can be rapidly resumed and, therefore, fit well for the purpose of fine tuning the timely controlled developmental processes.

**Figure 2:**
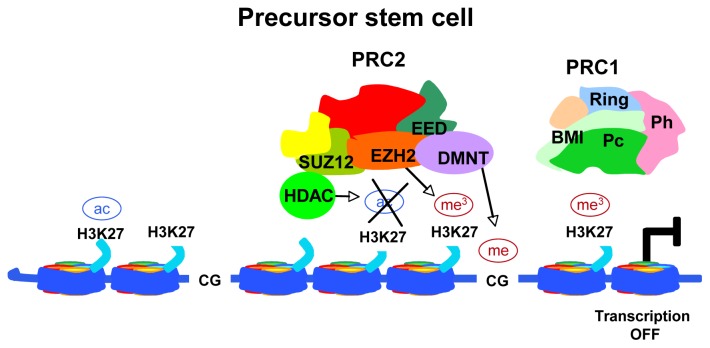
Schematic representation of transcriptional gene repression by PRC2. In an uncommitted stem cell, the core component EZH2 methylates histone H3 on K27, thus generating the epigenetic mark H3K27me3. The HDAC activity favors this EZH2 effect deacetylating H3K27. In this way, the PRC1 complex is recruited and binds to DNA, thus stabilizing the repressive state of the chromatin. The final result is the transcriptional repression of developmental genes.

DNA methylation is catalyzed by DNA methyltransferases (DNMTs) and results in *de novo* methylation of unmethylated DNA and/or methylation maintenance of hemimethylated sequences. In normal tissues, DNA methylation is typically present and stable in the intergenic regions. In cancer cells, DNA methylation is exclusively found at the level of cytosines within CpG-rich regions of gene promoters leading to gene silencing. On the contrary, DNA hypomethylation of these CpG islands, also often aberrant in cancer, can increase gene expression [10]. The final result is either the silencing of tumor suppressor genes or the transcriptional activation of proto-oncogenes. In addition, DNA hypomethylation can lead to DNA helix breakpoints and, ultimately, to loss of heterozygosity (LOH) or aberrant chromosomal rearrangements. In strict conjunction with these mechanisms, the histone code generated by covalent modifications on histone tails, regulates chromatin remodeling for the accessibility of the transcription machinery to genes up to DNA repair, replication and segregation [11]. The major modifications of histone tails are controlled by histone acetyltransferases (HAT) and histone demethylases (HDM) in a competitive manner with histone deacetylases (HDAC) and histone methyltransferases (HMT), respectively. Among HMTs, the Polycomb group (PcG) protein EZH2, a component of the Polycomb repressor complex 2 (PRC2), is responsible for the trimethylation of Lysine 27 on histone H3 (H3K27me3) on gene promoters (Figure 2).

This modification allows the recruitment of the PRC1 complex which inhibits gene transcription through histone H2A ubiquitination. HDACs participate to the PRC complexes reinforcing the inhibition of gene expression by deacetylating H3K27, thus favoring H3K27 trimethylation. Finally, PcG complexes recruit DNMTs to specific gene loci to induce transcriptional silencing through DNA methylation (Figure 2). The final result is the maintenance of cell stemness and the support of self-renewal and pluripotency. Consistently, components of the PRC complexes are often aberrantly over-expressed in tumors (reviewed in [12]). Of note, epigenetic modifications that are maintained through mitosis and inherited during cell development and differentiation can be reversed by treatment with appropriate drugs. Therefore, compounds acting on “epimutations” can be used in association with conventional chemotherapy to induce growth arrest, differentiation and tumor cell death.

## 3. The Small Non-Coding RNAs, “microRNAs”

MiRNAs are a class of non-coding ~19–22 nucleotides (nt) single-strand RNAs transcribed in a developmental and tissue-specific manner during embryogenesis [13]. More than 1500 mature miRNAs have been identified in humans (http://www.mirbase.org), which are highly conserved across species. miRNAs are involved in post-translational gene silencing by binding complementary sequences in the 3′-untraslated regions (*UTRs*) of a target mRNA through their “seed” sequence leading to translational repression or mRNA degradation [14,15].

The expression of more than 60% of human protein-coding genes is controlled by miRNAs (reviewed in [13]). Several miRNAs can target the same mRNA and, conversely, each miRNA can target several mRNAs leading to additional layers of post-transcriptional control of gene expression. To further expand the scenario, specific miRNAs have been recently described to upregulate gene transcription also in quiescent cells [16] and others to target sequences within the 5′UTR [17], exonic regions [18] or gene promoter regions [19].

**Figure 3:**
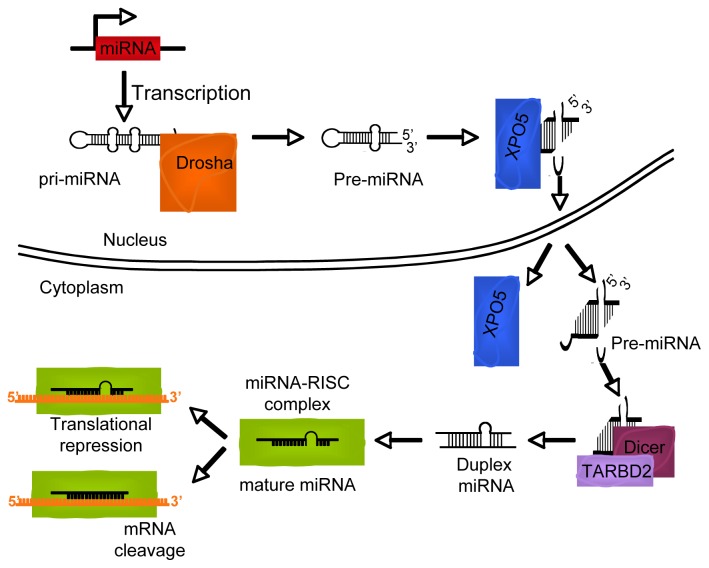
The biogenesis of miRNAs. MiRNAs are transcribed as longer RNA precursor (pri-miRNA) by RNA polymerase II and/or III and processed by the Drosha complex in pre-miRNAs. These fragments are exported from the nucleus by exportin 5 (XPO5). In the cytoplasm pre-miRNA are further processed by Dicer and TAR RNA-binding protein 2 (TARBD2) to generate mature miRNAs, which are loaded into the RNA-induced silencing complex (RISC). The translation of mRNAs targets into proteins is repressed by mRNA degradation or, alternatively, block of mRNA translation.

Mature miRNAs are the result of sequential processing of longer RNA precursors transcribed by RNA polymerase II and/or III and called pri-miRNA (Figure 3) [20,21]. This long transcript is processed by Drosha, a ribonuclease-III protein, in collaboration with DGCR8, a protein responsible for anchoring the pri-miRNA into the complex. The resulting 70-nt-long molecule termed pre-miRNA, is exported to the cytoplasm by the nuclear export protein Exportin-5 [13,22]. In the cytoplasm, the pre-miRNA is released and processed by Dicer to produce the functional ~19–22-nt double-strand RNAs. The less stable single strand is loaded by the Dicer-TRPB complex into the RNA-induced silencing complex (RISC) which carries it to complementary 3′UTR mRNA sequences.

MiRNAs regulate fundamental physiologic processes such as embryonic development, lineage/tissue identity specification and homeostasis [23–27]. Therefore, it is not surprising that miRNAs display an aberrant expression profile in a wide range of human diseases [13]. In tumor cells, as reported for protein-coding gene, miRNA promoters can be aberrantly modified by the deregulated epigenetic machinery leading to changes in their expression profile [28–31]. Increasing evidence suggesting tumor suppressor or oncogenic role for miRNAs has been obtained in adult cancers [1,13,32–34]. Consistently, the re-expression of tumor suppressor miRNAs in tumor cells has been suggested as a potential anti-cancer therapeutic approach [35–40].

Recently, miRNAs have been shown, by us and other groups, to play a role also in the pathogenesis of embryonal pediatric cancers such as RMS and NB [41–43]. So far, the majority of tumor suppressor miRNAs involved in RMS and NB regulates the differentiation of skeletal muscle and neuronal compartments, respectively. These miRNAs are often silenced by aberrant DNA or histone methylation or located in unstable chromosomal regions often involved in LOH, ie miR-34a [13,44].

Consistently, their re-expression after treatment with HDAC inhibitors, de-methylating compounds or differentiating agents is sufficient to allow tumor cell differentiation and to impair tumorigenesis. Therefore, an epigenetic approach aimed at re-expressing these miRNAs could have a therapeutic value.

## 4. Rhabdomyosarcoma

RMS is a skeletal muscle-derived tumor and the most common soft-tissue sarcoma of childhood [45]. RMS cells express transcription factors specific to skeletal muscle progenitors, such as Myogenic Differentiation (MyoD) and myogenin [46,47], but their intrinsic myogenic program is disrupted at different stages of differentiation. In line with these observations, restoring terminal differentiation in RMS cells leads to inhibition of cell proliferation *in vitro* and tumor growth *in vivo*.

Pediatric RMS includes two major histological subtypes, namely alveolar (~25% of cases) and embryonal (~75% of cases) RMS [45,48]. The five-year overall survival rate of non-metastatic embryonal RMS patients is around 70%, while that of alveolar RMS and metastatic patients is still in the order of 25%, supporting the urgent need of novel therapeutic approaches.

The majority of alveolar RMS are characterized by chromosomal translocations, such as t (2; 13) or t (1; 13), leading to the expression of PAX3/FOXO1 or PAX7/FOXO1 fusion proteins, respectively [49–51]. These fusion products, especially PAX3/FOXO1, are a hallmark of high-risk tumors and correlate with poor prognosis [52]. Approximately 20% of alveolar RMS do not present known chromosomal translocations and are molecularly and clinically indistinguishable from embryonal ones [53–55]. However, novel chromosomal translocations involving PAX3 have been recently discovered in a subset of alveolar RMS previously diagnosed as fusion negative [51,56].

During skeletal muscle tissue development, PAX3 expression is detected in pluripotent progenitors and is followed by that of PAX7, which characterizes myogenic committed cells [57,58]. These transcription factors, on one hand, induce the expression of MyoD that marks myoblasts, and on the other hand, stimulate self-renewal and proliferation of myogenic cells. Thus, their expression is fundamental to assure the balance between cell proliferation and differentiation during embryogenesis leading to the right number of committed progenitors in order to form multinucleated myofibers. During differentiation, pro-myogenic miRNAs downregulate PAX3 and PAX7 to achieve complete myogenesis (reviewed in [59]).

An altered expression of miRNAs acting as pro-myogenic regulators has been reported in RMS, [43,59–61]. These miRNAs behave as tumor suppressors when re-expressed, halting tumorigenic processes working in negative feedback circuitries with epigenetic regulators. These findings demonstrate that aberrant epigenetic control of miRNA expression concurs to RMS pathogenesis.

### 4.1. Epigenetically Deregulated miRNAs in Rhabdomyosarcoma

Muscle-specific miRNAs that regulate myogenesis are termed “myomiRs.” To this group belong three miRNA clusters, miR-1-1/miR-133a-2, miR-1-2/miR133a-1 and miR-206/miR-133b, encoded by three bicistronic miRNA genes on separate chromosomes (reviewed in [62]).

**Figure 4:**
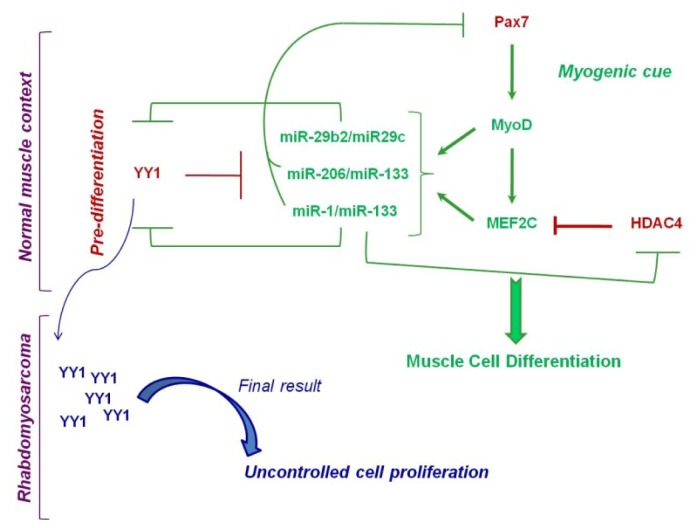
Circuits involving the Polycomb Group (PcG) protein YY1 and miRNAs in normal and Rhabdomyosarcoma contexts. During myoblasts expansion, YY1 represses miR-29, miR-1 and miR-206 cluster expression. Under a myogenic stimulus, Pax7 induces the muscle regulatory factor MyoD that allows MEF2C activation, leading to miR-29, miR-1 and miR-206 de-repression. MiR-1 and miR-29 clusters post-transcriptionally block YY1 expression. Moreover, downregulation of Pax7 caused by miR-1 and miR-206 and that of HDAC4 caused by miR-1 ultimately leads to differentiation of myogenic precursors. In the RMS context, YY1 expression is highly deregulated resulting in pro-myogenic miRNAs repression that causes uncontrolled cell proliferation and muscle differentiation impairment.

Although no direct epigenetic regulation of the expression of miR-1 and miR-206 clusters has been highlighted in RMS, a recent study provided several evidences linking these miRNAs to the epigenetic machinery in normal myoblasts [63]. In this work, YY1 was shown to regulate the expression of miR-1 and miR-206 clusters in murine myoblasts *in vitro* and *in vivo*. In particular, the authors found YY1 binding sites in previously identified muscle-specific enhancers [64] located (i) upstream of miR-1-2 (E1) and (ii) in an intron between miR-1-2 and miR-133a-1 (E2), and (iii) between miR-1-1 and miR-133a-2 gene loci (E3). Moreover, they discovered a previously unknown enhancer upstream of miR-206 and miR-133b coding regions (E4) showing YY1 binding site. They demonstrated that YY1 repressed the activity of all these four enhancers impairing the expression of miRNAs [63]. Interestingly, Ezh2 was present and active on the enhancers E1 and E3 but not on E2 and E4 suggesting that YY1 can function in myoblasts in a HMT-independent manner. Furthermore, the same authors uncovered a feedback loop between miR-1 and YY1 demonstrating that miR-1 directly targets the 3′UTR of YY1 mRNA. Having demonstrated that miR-1 is able to target PAX7, as already reported for miR-206 [65], which is consistently upregulated in YY1 over-expressing myoblasts, the authors depicted an anti-myogenic network in which YY1 plays a central role in repressing miR-1/miR-206 (Figure 4). This network is flexible and bi-univocal due to the feedback control of miR-1 on YY1 expression [63].

Additional links with epigenetic networks in myoblasts have been shown for both miR-1 and miR-206 clusters. The ectopic expression of these miRNAs inhibits HDAC4, which sustains cell proliferation by preventing the expression of the cyclin-dependent kinase inhibitor p21^Cip1^, essential for terminal muscle differentiation [66,67]. This finding is of interest for the RMS context due to the potentiality of therapeutic approaches with HDACs inhibitors [68,69]. Interestingly, miR-29b directly targets HDAC4 during osteoblast differentiation [70], suggesting an intricate complex of miRNAs pathways acting similarly in different tissue contexts. Collectively, these observations suggest that miRNAs can influence gene transcription through the control of several types of epigenetic repressors. Of note, crucial components of molecular pathways related to RMS aggressiveness, such as IGF and RAS, can be post-transcriptionally downregulated by pro-myogenic miRNAs. Indeed, (i) miR-1 and miR-133 target IGF receptor 1, thus avoiding aberrant muscle hypertrophy [71,72]; (ii) miR-214, which is repressed by EZH2 in proliferating myoblasts, is able to favor myogenesis by downregulating the anti-myogenic N-RAS oncogene [73,74].

In addition to myomiRs, there are non-muscle-specific miRNAs that participate to skeletal muscle differentiation such as miR-181a/miR-181b, miR-27a, miR-27b, miR-26a and miR-29b2/miR-29c. Wang and colleagues provided the first evidence of an epigenetic deregulation of miRNAs in RMS discovering a regulatory circuitry between miR-29b2/miR-29c and the PcG protein YY1 [60]. The same authors had previously demonstrated that during the expansion of myogenic precursors, NF-kB inhibited terminal differentiation maintaining a high level of YY1, which, in turn, repressed the expression of myofibrillary genes favoring cell precursor proliferation [75]. Therefore, reasoning that miRNAs are regulators of myogenesis, the authors investigated whether the anti-differentiation effect of YY1 was at least in part related to the repression of pro-myogenic miRNAs [60]. Indeed, they identified miR-29b2/miR-29c cluster on chromosome 1 being repressed by YY1 in proliferating myoblasts (Figure 4). This repression involved the recruitment of both HDAC1 and the EZH2 PcG protein, which deacetylated and trimethylated, respectively, the Lys 27 on histone H3 in a highly conserved region 20 kb upstream of the miR-29b2/miR-29c gene locus. Under myogenic cues, miR-29b2/miR-29c gene cluster is expressed and directly targeted YY1 reinforcing myogenesis. Of note, in myogenic precursors undergoing early-stage differentiation, YY1 and EZH2 have been shown to work in concert to repress the transcription of late-stage muscle-specific genes such as Muscle Creatine Kinase (MCK) and Myosin Heavy Chain (MHC) to avoid premature differentiation [76]. Therefore, a fine tuning between anti- and pro-myogenic molecular stimuli underlies a proper myogenesis during which both epigenetic and miRNAs pathways appeared highly interconnected.

The miR-29b2/miR-29c-YY1 epigenetic negative feedback circuitry [60] was shown by the authors to be disrupted in RMS cells, in which YY1 and EZH2 over-expression resulted in persistent activation of stemness maintenance program. The tumor suppressor role of miR-29b2/miR-29c was clearly evidenced by gain-of-function experiments demonstrating that forced over-expression of this miRNA cluster in RMS cells is sufficient to impair the tumorigenic properties both *in vitro* and *in vivo* by repressing YY1 expression. Consistent with these observations, tumor tissues from RMS patients showed upregulation of YY1 and EZH2 [60]. In line with this report, preliminary results from our laboratory showed that EZH2 downregulation in RMS cells impairs tumorigenesis, thereby allowing the de-repression of several tumor suppressor miRNAs, including the miR-29b2/miR-29c cluster.

Interestingly, even if YY1 binding sites were not found in the promoter of miR-29a/miR-29b1, this miRNA cluster appeared downregulated in primary sarcoma samples as compared to skeletal muscle tissues [77]. Interestingly, the miR-29 family has been shown to directly target DNMT3A and DNMT3B in several types of cancer, thus suggesting a link between their reduced expression and pathological gene hyper-methylation [78,79].

Altogether, these results highlight the importance of bi-univocal regulation of epigenetic molecules and miRNAs in skeletal muscle differentiation that should be considered also in the context of RMS in which these pathways are deregulated.

## 5. Neuroblastoma

NB is a neuroectodermal tumor that originates from precursor cells of the sympathetic nervous system and represents the third leading cause of cancer-related death in childhood [80]. The heterogeneous clinical behavior, ranging from spontaneous regression to rapid progression, is attributable to biological and genetic characteristics of the tumor.

NB diagnosed in patients under one year of age usually has a favorable prognosis since tumor cells undergo spontaneous differentiation or regression, whereas NB occurring in patients over one year of age tend to grow aggressively resulting in a fatal outcome. The prognosis of stage I-III NB, with a tumor confined to the originating organ or surrounding tissue, is quite favorable, whereas that of stage IV NB, where the tumor is metastatic, is dismal. Stage IV-S NB is a metastatic disease seen exclusively in infants, which is associated with high survival rate due to the spontaneous maturation and regression of tumor cells. Nevertheless, since disease and risk staging are not comprehensive and fully precise, they should be considered as surrogate markers of the underlying tumor biology.

NB cells correspond to adrenal neuroblasts arrested at different stages of sympathoadrenal development, thus representing multipotent progenitor cells with specific tumorigenic potential [81,82]. The degree of differentiation of NB is another important factor for establishing prognosis. Based on this, NB tumors are classified in different risk categories spanning from more mature and benign ganglioneuromas (GN), to intermediate and potentially malignant ganglioneuroblastomas (GNB), to undifferentiated NB, always malignant with worst prognosis. The factors responsible for malignant transformation from sympathetic neuroblasts into neuroblastoma cells are not well understood. In several NB cell lines, all-*trans* retinoic acid (ATRA)-induced differentiation is associated with increased expression of neurotrophic receptors, including Trk family receptors and glial cell-derived neurotrophic factor receptor, Ret. Of note, ATRA is a compound related to 13-cis-retinoic acid that is used as differentiating agent in children with high-risk NB.

From a clinical point of view, the presence of any segmental chromosomal imbalances correlates with a more aggressive phenotype, whereas tumors containing mostly whole chromosome gains or losses are associated with a benign phenotype and high propensity for spontaneous regression or differentiation. The most widely characterized cytogenetic alterations in NB tumors include amplification of the *MYCN* oncogenic transcription factor at chromosome band 2p24, LOH or rearrangements of the distal portion of the short arm of chromosome 1 (1p31-term), chromosome 3 (3p22) and chromosome 11 (11q23), or gains of chromosome arm 1q or 17q. Besides these chromosomal/molecular abnormalities, gains of chromosomes 4q, 6p, 7q, 11q and 18q, amplification of *MDM2* and *MYC* genes and *LOH* at 14q, 10q and 19q13 have also been described [83]. In particular, loss of chromosome 1p region, occurring mainly through an unbalanced translocation that results in the gain of 17q [84], arises preferentially in tumors with *MYCN* amplification [85]. Some of these genetic alterations were proven to be independently associated with a poor clinical outcome. The region of 1p, lost in NB tumors, is quite large and contains multiple genes that have been shown to contribute to NB pathogenesis [86,87]. Similarly, the minimal common region of gain at distal 17q23 has been shown to contain at least 15 genes that remain to be investigated [88]. Of note, *MYCN* is one of the major players in normal neural crest differentiation, suggesting that its amplification/deregulation is the result of a developmental defect that probably has occurred during embryonic development [89]. However, although *MYCN* amplification is considered the most important prognostic marker of highly aggressive tumors; it is present in less than 30% of NB tumors [90].

More recently, activating mutations in the anaplastic lymphoma kinase (*ALK*) gene have been associated with the majority of hereditary NB and a 10% of sporadic NB cases [91–94]. ALK is a receptor tyrosine kinase involved in several other human cancers, including anaplastic large-cell lymphoma and non-small cell lung cancer [95–97]. ALK signaling drives cell transformation *in vitro* and *in vivo* through several pathways such as cell-cycle, survival and cell migration [98,99]. Nevertheless, additional molecular alterations concur to NB pathogenesis and their identification is essential to improve the prognostic classification of NB patients.

In the attempt to improve the risk stratification of NB subtypes, several groups have characterized and correlated methylation profiling of biologically and clinically different subgroups of NB patients with clinical risk factors and survival (for a detailed review see [100]). The first DNA methylation studies in NB have revealed that silencing of caspase 8 and RAS-association domain family 1 isoform A (RASSF1A) are important in the development and progression of disease [101,102]. Both genes are often methylated in primary NB cells and their methylation status negatively correlates with survival.

Recent advances in genome-wide methylation screening methodologies, such as re-expression analysis after treatment with 5-aza-2′-deoxycytidine (DAC), DNA methylation promoter assay after affinity-based capture, methylation microarray after bisulfate treatment and next-generation sequencing techniques, have allowed the identification of 75 different DNA methylation biomarkers involved in fundamental biological processes operative in NB ([103–105] and reviewed in [100]).

Epigenetic inactivation of miRNAs with tumor-suppressor activities is also recognized as a major hallmark of NB tumors. A recent study from Chavali group [106] showed that the majority of the miRNA genes are flanked by scaffold/matrix-attachment region (S/MAR) elements that regulate their tissue and cell type-specific expression by binding to epigenetic regulators MAR binding proteins. In this study, the authors showed that the miR-17-92 cluster, a well-known example of oncomiR that is over-expressed in high-risk NB [41], has two conserved MARs elements, one upstream and one downstream the cluster, being the former strongly bound by HMG I/Y protein, a chromatin modulator that promotes an open conformation facilitating mRNA transcription. Consistent with this finding, the miR-17-92 cluster expression was down-regulated in cells interfered for HMG I/Y family expression [106].

### 5.1. Epigenetically Deregulated miRNAs in Neuroblastoma

Recent studies from Stallings’ group revealed extensive epigenomic changes in NB cells upon ATRA exposure, a phenomenon that is associated with a complex series of molecular events, including modifications of both chromatin compaction and DNA methylation status [107]. Intriguingly, in a previous paper, Das and colleagues showed that several miRNAs that are upmodulated as a consequence of ATRA treatment target DNA methyltransferases involved in the process of ATRA-induced differentiation of NB cell lines [108]. More recently, the same authors revealed that DNA methylation changes occurred during ATRA treatment include several regulatory regions of tumor suppressor miRNAs [109]. The authors identified 67 miRNAs sensitive to the effects of DNA methylation in 18 primary NB tumors and four NB cell lines by the combined use of methylated DNA immunoprecipitation, miRNA and mRNA target analysis. Of note, a relative high proportion of these epigenetically silenced miRNAs (42%) were significantly associated with poor patient survival when under-expressed in tumors, whereas other 10 miRNA (15%) were found hyper-methylated in favorable tumors and over-expressed in tumors from patients with poor survival. Remarkably, not only miRNAs, but also their predicted gene targets, were significantly associated with poor patient survival when over-expressed in tumors. In particular, miRNA-340 was functionally inactivated by DNA methylation, re-expressed in NB cell lines following DAC treatment and significantly associated with poor survival when under-expressed in primary NB tumors. According to the NB cell line used, over-expression of miR-340 induced either apoptosis or differentiation by targeting SOX2, an SRY box containing a transcription factor family member that acts in regulating the maintenance and differentiation of neural stem cells [109].

**Figure 5:**
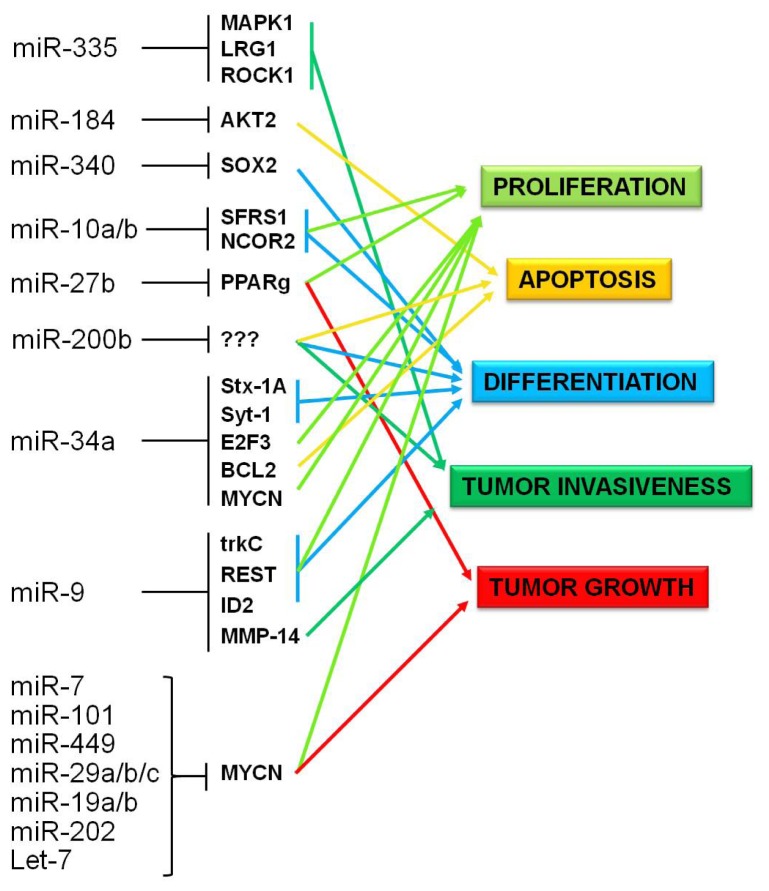
Scheme of cellular pathways affected by methylation-sensitive miRNAs in Neuroblastoma. Experimental validated gene targets and pathways affected by altered expression of methylation-sensitive miRNAs are indicated.

Several of the epigenetically regulated miRNAs identified in this integrated analysis were previously found to negatively impact NB growth both *in vivo* and *in vitro* (Figure 5). The main findings regarding their implications in NB pathogenesis are described as follows.

MiR-10a and -10b were found to induce NB differentiation through multiple gene targeting (for a detailed review see [110]). Meseguer *et al.* demonstrated that these microRNAs directly target the SR-family splicing factor (SFRS1), a regulator of alternative splicing that plays a role also in enhancing translation initiation of mRNAs containing specific SFRS1 binding sequences [111]. Of note, this factor seems to be a proto-oncogene deregulated in many forms of tumor [112]. Foley and colleagues identified the nuclear receptor co-repressor 2 (NCOR2) as the primary target of miR-10a and -10b responsible for causing differentiation [113]. NCOR2 is a transcriptional co-repressor that recruits a complex of proteins that include SIN3A/B and histone deacetylases HDAC1, HDAC2 and HDAC3 to DNA promoter regions. Interestingly, neural stem cells from mice lacking NCOR2 exhibit extensive neurite outgrowth and reduction in cell proliferation [114]. More intriguingly, siRNA-mediated inhibition of NCOR2 resulted in a significant increase in miR-10a level that suggests a possible regulatory feedback loop in the differentiation process [113].

A number of these epigenetically regulated miRNAs were previously found to be involved in a complex gene network involving MYCN. A study from Buechner and colleagues demonstrated that the tumor suppressor miRNAs let-7 and miR-101 inhibit proliferation and clonogenic growth of Kelly NB cell line by targeting MYCN [115]. Furthermore, they experimentally validate miR-449, miR-19a/b, miR-29a/b/c, and miR-202 as direct MYCN-targeting miRNAs. A very recent paper by Molenaar and colleagues, elegantly demonstrated the existence of a LIN28B-let-7-MYCN axis in NB [116]. Using a mouse model with neural crest-specific over-expression of LIN28B, these authors showed that LIN28B drives NB upregulating MYCN protein through let-7 repression. Notably, MYCN has been recently discovered to bind and transcriptionally downregulate another epigenetically controlled miRNA, miR-335, which in turn regulates genes in the TGF-β non-canonical pathway, such as the Rho-associated coiled-coil containing protein (ROCK1), MAPK1 and putative member LRG1, leading to inhibition of invasiveness and migratory potential of NB cells [117]. The same group had previously discovered a pro-apoptotic effect of miR-184 both *in vitro* and *in vivo*, through the direct targeting of AKT2, a serine/threonine kinase active downstream of the PI3K pathway. Interestingly, MYCN could exert its tumorigenic effect also by suppressing either directly or indirectly the same miRNA [118,119].

Several groups identified miR-542-5p as a putative tumor suppressor, whose expression has a prognostic value and might be important in NB tumor biology [120–122]. Of note, miR-542-5p is predicted to target TBR1, a gene essential for proper development of neurons. Functional studies that address this or other putative targets should be performed in the future.

The brain specific miR-9 was demonstrated by Laneve *et al.* to play an important role in controlling NB cell proliferation through a regulatory circuit involving TRKc and other two miRs, miR-125a and - 125b [123]. The same authors discovered a regulatory feedback mechanism involving miR-9 and the neuro-restrictive silencer factor REST, suggesting a fine interplay for the maintenance of the neuronal differentiation program [124]. More recently, Nasi and colleagues found that the transcription factor ID2, which is involved in the self-renewal and proliferation of neural precursor cells, is a target of miR-9 [125]. At the same time, another group demonstrated that miR-9 reduced matrix metalloproteinase (MMP)-14 level by inhibiting the invasion, metastasis, and angiogenesis of SH-SY5Y and SK-N-SH NB cell lines both *in vitro* and *in vivo*[126].

Lee and colleagues showed that the epigenetically regulated miR-27b acts as a tumor suppressor in NB by inhibiting the tumor-promoting function of peroxisome proliferators-activated receptor (PPAR)y and blocking cell growth *in vitro* and tumor growth in mouse xenografts [127]. In addition, miR-27b indirectly regulates also NF-kB activity and transcription of inflammatory target genes, which triggers an increased inflammatory response.

As described above, LOH of 1p36 is commonly found in NB with *MYCN* amplification and is associated with poor outcome. Of note, five microRNAs (miR-200a, miR-200b, miR-429, miR-34a and miR-551a) map within the first 10Mb on chromosome 1 short arm (1p36.22 to 1pter), suggesting that the loss of these miRNAs could contribute to the acquisition of an aggressive NB phenotype.

MiR-200b was originally identified through a positional gene candidate approach looking for miRNAs that map within chromosomal regions frequently altered in NB [128]. Conversely, Ragusa and colleagues discovered that the miR-200b region is unaffected by the frequent deletions involving 1p36 in the NB cell lines analyzed [129]. Moreover, they found a CpG island near the gene-encoding miR-200b and showed that, upon treatment with demethylating agents, miR-200b levels were upregulated in six NB cell lines. Finally, they provided several evidences supporting a role for miR-200b in NB cell invasiveness, differentiation and apoptosis.

MiR-34a expression is silenced in several types of cancer due to aberrant CpG methylation of its promoter [130]. In NB cell lines and primary NB tumors, miR-34a is absent or expressed at low levels, while it is upregulated in a dose-dependent manner following ATRA treatment [131]. Given that miR-34a has been implicated in NB growth inhibition, and mutations of the retained allele are not a common mechanism towards achieving biallelic inactivation, it is plausible that other mechanisms such as epigenetic regulation could contribute for loss of miR-34a expression in NB. Of note, miR-34a expression has been shown to increase after HDAC inhibition in a bladder cancer cell line [132]. It will be of interest to validate this finding also in the NB context since several studies demonstrated the involvement of this microRNA in NB pathogenesis. Welch and colleagues demonstrated a tumor suppressive role for miR-34a through the transcription factor E2F3 targeting [131]. Moreover, Wei *et al.* provided evidence that MYCN is a direct target of miR-34a, as further confirmed by a functional screen study by Cole *et al.*, which also demonstrated a BCL2 direct targeting [128,133]. Recently, synaptotagmin I (Syt-I) and syntaxin 1A (Stx-1A), two proteins involved in synaptogenesis and neuronal differentiation, were found to be targets of miR-34a [134].

All these findings reveal a network of epigenetic pathways and miRNA interactions whose dysregulation contributes to NB, and that has translational consequences for the management of the disease.

## 6. Translational Perspectives and Conclusions

The majority of miRNAs aberrantly downregulated in RMS and NB behave, when re-expressed, as tumor suppressors re-establishing a proper differentiation program. Therefore, they represent promising therapeutic targets. Moreover, miRNAs detection in cancer patients could represent a powerful prognostic and diagnostic tool. A miRNA signature has been recently reported for RMS primary samples by two groups [77,135]. Intriguingly, the first group suggested that an embryonal sample had been previously misdiagnosed allowing a re-evaluation of the tumor followed by a diagnosis as alveolar [77]. The second group showed that miRNA expression clusterings in RMS samples are correlated with PAX3/FOXO1, PAX7/FOXO1 and embryonal subgroups, and, thus, are potentially useful for risk stratification [135].

Recently, miRNA signatures have been shown to be related also to NB prognosis. Lin and colleagues found that, using a combination of 15 biomarkers that consist of 12 miRNAs’ signature, expression levels of Dicer and Drosha and age at diagnosis, it was possible to segregate 66 NB patients into four distinct patterns that were highly predictive of clinical outcome [136]. Subsequently, a signature of 25 miRNAs was built and validated on an independent set of 304 frozen tumor samples and 75 archived formalin-fixed paraffin-embedded samples. This signature was significantly able to discriminate the patients with respect to progression-free and overall survival [137].

Furthermore, the possibility to detect and quantify miRNAs in body fluids whose profile mirrors physiological and pathological condition could also represent a reliable method to improve prognosis and diagnosis [52,138,139].

MiRNAs re-expression in tumors rises doubts regarding delivery, efficacy and safety of these molecules *in vivo*. Viral and non-viral vectors have shown pre-clinical feasibility and efficacy in animal models including primates [36,140–145]. However, their application on human patients could induce side effects linked to their immunogenicity or could be ineffective due to lack of specificity (reviewed in [146]). The use of nanoparticles represents an efficient system to deliver cDNA to cells and, therefore, it could be potentially useful for the delivery of miRNAs *in vivo*. Indeed, a first promising study using nanoparticles encapsulating miR-34a and conjugated to a GD2 antibody demonstrate the feasibility of a targeted delivery miRNA-based therapy for NB [147].

Based on these observations, therapeutic approaches able to modulate miRNA gene expression targeting epigenetic regulators present several advantages. Conversely to DNA mutations, epigenetic modifications are reversible and responsive to specific compounds, which gave promising results in clinical trials for adult tumors. Indeed, low doses of DNA methyltransferases inhibitors azacitidine (AZA) and DAC have shown therapeutic effects in myelodysplasia/leukemia as well as in lung cancer patients [148–153]. It is noteworthy that older trials with high doses of epigenetic inhibitors have highlighted extreme toxicities and no effect on epigenome [154,155]. Very recently, an elegant work of Tsai *et al.*, investigated the mechanisms involved in the efficacy of low nanomolar doses of DAC and AZA in preclinical models of acute myeloid leukemia, breast, colon and lung cancers [156]. The authors showed that high-dose treatments resulted in high cytotoxic effects on tumor cells. Nevertheless, residual cells engrafted in immunodeficient mice were still able to grow and give tumors. Conversely, low concentrations of AZA or DAC reduced self-renewal of tumor cells both *in vitro* and *in vivo,* sustaining global DNA demethylation and re-expression of anti-tumor master gene pathways [156]. These results strongly confirm the potential efficacy of an epigenetic therapy that could re-express tumor suppressor genes and miRNAs.

Moreover, epigenetic targeted therapy with inhibitors of both DNA methyltransferases and HDACs, has shown anti-tumor effectiveness combined with a restored expression of tumor suppressor genes in preclinical models [157–159]. More important, well-tolerated combination regimens for low-dose AZA and HDAC inhibitors have been found effective in patients with myelodysplasia/leukemia [160,161]. Finally, very encouraging results have been reported in a phase I/II clinical trial on the use of a combined epigenetic low-dose therapy in recurrent metastatic non-small cell lung (NSCL) cancer refractory to conventional therapy [153]. In brief, AZA and entinostat, an inhibitor that blocks HDAC1-3 and HDAC9 function, were used on 45 NSCL patients, resulting in limited side effects while having efficacy on the aberrant epigenome of tumor cells. These findings suggest the potential use of these compounds also in solid cancers.

Novel agents that inhibit histone methyltransferases among which EZH2 are of particular interest for future applications since they do not require cell division to target cancer cells [162,163]. These compounds have been shown to synergize with other epigenetic agents in preclinical studies, representing promising drugs in order to de-repress EZH2 targeted miRNAs [164,165].

However, as reported for other targeted therapies, escape strategies for the miRNA re-expression approach could select resistant cells expressing mRNAs with mutated miRNA binding sites in their 3′UTRs. Moreover, PAX3/or PAX7/FOXO1 mRNAs expressed in fusion-positive alveolar RMS, being devoid of 3′UTR regions of PAX genes, are resistant to miRNAs’ post-transcriptional regulation. In addition, the de-repression of oncogenes could be a potential side effect of an epigenetic therapy [166]. Therefore, although the field of investigation in the epigenetic treatment of cancer appears very promising, further studies are needed to enhance the knowledge on the epigenome and its regulation both in normal and tumor contexts.
